# Supplementary material for: The effect of echoes interference on phonon attenuation in a nanophononic membrane
Source: Nat Commun. 2024 Feb 13;15:1317. doi: 10.1038/s41467-024-45571-x (PMC10864405; doi:10.1038/s41467-024-45571-x)
Supplement: Supplementary file 1 — Supplementary Information [file 41467_2024_45571_MOESM1_ESM.pdf]

Supplementary material: The effect of echoes interference on phonon attenuation in a nanophononic membrane

Mohammad Hadi,[1] Haoming Luo,[1,2,3] Stéphane Pailhès,[1] Anne Tanguy,[2] Anthony Gravouil,[2] Flavio Capotondi,[4] Dario De Angelis,[4] Danny Fainozzi,[4] Laura Foglia,[4] Riccardo Mincigrucci,[4] Ettore Paltanin,[4] Emanuele Pedersoli,[4] Jacopo S. Pelli-Cresi,[4] Filippo Bencivenga,[4] and Valentina M. Giordano[1,*]

[1]*Institute of Light and Matter, UMR5306 Université Lyon 1-CNRS, Université de Lyon, F-69622 Villeurbanne cedex, France*
[2]*LaMCos, INSA-Lyon, CNRS UMR5259, Université de Lyon, F-69621 Villeurbanne Cedex, France*
[3]*LMS, CNRS, École Polytechnique, Institut Polytechnique de Paris, 91128 Palaiseau, France*
[4]*Elettra Sincrotrone Trieste S.c.P.A., Strada Statale 14, km 163.5, AREA Science Park, I-34149 Basovizza, Trieste, Italy*

(Dated: January 24, 2024)

I. EUV TG SETUP

EUV TG experiments were carried out at the TIMER beamline using a double stage FEL source (FERMI FEL2), which was set in order to have: i) a costant wavelength output from the second stage (λ_{pr} =13.3 nm), that was used as a probe, and ii) a variable and selectable wavelength output from the first stage, corresponding to sub harmonics of the second stage, that was used as a pump (λ_{ex} =26.6, 39.9 or 53.2 nm). The estimated pulse duration was $\approx$ 40 fs full width at half maximum (FWHM) for the probe and $\approx$ 50-60 fs FWHM for the pump, with a neglible jitter between them. The polarization of both pulses at the source was circular, the pulse energy in the 10-100 μJ range and the spectrum almost Fourier transform limited. The FEL intensity was monitored on a shot-to-shot basis by two gas monitors and a spectrometer, which also provided a shot-to-shot determination of the probe's spectrum. The combined information from spectrometer and gas monitors allowed to determine the pulse energy of both pump and probe pulses.

The FEL beam was then split into three parts by wavefront division plane mirrors and routed into three different branchlines, one of them was equipped with a right angle delay stage based on 4 multilayer (ML) mirrors with high reflectivity at λ_{pr}. An active feedback permitted to keep stable the probe's spot at the sample position during the Δt scans. The 4 ML reflections are also act as spectral filtering, permitting to effficiently reject (extinction factor $> 10^8$) the unwanted radiation at λ_{ex} from the probe's branchline. The unwanted radiation at λ_{pr} was removed from the pump branchlines by thin aluminum foils. The three beams are thus focused at the sample position at the desired angle by three focusing toroidal mirrors; the three beams lye in the same (horizontal) plane.

The crossing angle of the two pump beams was 2θ =27.6°, with the sample surface orthogonal to their bisector, while the angle of incidence of the probe beam was 4.6° with respect to the normal of the sample surface. This geometry correspond to the Bragg angle for the probe's transient diffraction when $\lambda_{ex}/\lambda_{pr}$ =3, which corresponds to one of the used experimental conditions. When $\lambda_{ex}/\lambda_{pr}$ $\neq$3 the Bragg conditions are not satisfied and, consequently, the efficiency of the EUV TG process decreases. However, in light of the short absorption length of the pump ($L_{abs} \leq 54$ nm) and the employed range in $L_{TG} \geq 56$ nm, such a decrease in efficiency was within an ecceptable level (> 10 %) [1]. The spot size on the sample was 300x280μm^2 FWHM and 280x230μm^2 FWHM for the two pumps and 450x330μm^2 FWHM for the probe. The mismatch between spot sizes is not critical as long as the fluence level required by the experiment can be achieved, since the EUV TG signal arises from the overlap region and propagates in a background free direction. The transmission of each pump breanchline was 0.04, 0.015 and 0.006 (including the aluminum filter) at λ_{ex} =26.6, 39.9 and 53.2 nm, respectively, resulting a fluence range $F = 0.12 - 0.34$ mJ/cm^2. We did not observe any appreciable sample damage, also after prolonged illumination (several hours). The EUV TG signal was finally detected by a CCD camera (PI-MTE), equipped with a light-tight zirconium filter to reject room-light that may leak into the experimental chamber and spurious EUV light at λ_{ex}, which may arise from diffuse scattering from the sample and stray-light from the beamline. To further reduce background around the signal, we introduced a beamstop to create a shadow on that region of the CCD. The FEL photon transport, sample environment and detection was in high vacuum in order to allow propagation of EUV beams; further details on the setup can be found elsewhere [2].

The main experimental parameters are summarized in Supplementary Table I.

* To whom correspondence should be addressed: valentina.giordano@univ-lyon1.fr

Sample	$\mathbf{L_{TG}}$(nm)	$\boldsymbol{\lambda_{ex}}$(nm)	$\mathbf{L_{abs}}$(nm)	F(mJ/cm^2)	T(C)
U	55.8	26.6	54	0.17(3)	35(2)
U	83.7	39.9	26	0.15(3)	37(2)
U	109.7	52.3	10	0.27(5)	50(5)
NS	55.8	26.6	54	0.24(5)	40(3)
NS	83.7	39.9	26	0.12(2)	35(2)
NS	109.7	52.3	10	0.27(5)	56(6)

Supplementary Table I. **Experimental parameters:** U stands for uniform sample, while NS for the nanostructured one. The values of L_{TG}, λ_{ex}, L_{abs} are given together with the F and the estimated values of T_s. Details and more parameters can be found in the Supplementary Material

II. TG SIGNAL FIT

As described in the main text, the signal is the superposition of a relaxing thermal signal and coherent phonon oscillations. The number of phonon modes is not fixed, as the EUV TG can in pronciple excite all phonons that can couple with its wavevector. In order to identify case by case the number of excited modes, we have performed the Fourier transform prior to any fit. Supplementary Fig. 1 reports the FFT for our experimental TG periods in both samples. At λ_{ph} = 106.7 nm, in both samples a single peak at around 100GHz was identified, together with its overtone at about 200 GHz. 2 peaks can be identified at λ_{ph} = 83.7 nm in both samples and at λ_{ph} = 55.8 nm in the uniform membrane, while in the waveform of the nanostructured sample at λ_{ph} = 55.8 nm, one can identifty 6 peaks (see vertical segments in the figure). However, a good fit can be obtained using 5 out of these 6 peaks.

Once the possible phonon modes are identified, the fit is performed with the function reported in the main text (Eq. 1). Supplementary Table II summarizes all fitting parameters for all EUV TG waveforms. Thermal parameters (A_{th} and τ_{th}) are reported only once for a given sample and value of L_{TG}, since they do not depend on the phonon branch. Supplementary Fig.2 reports the values of τ, as obtained from the fitting procedure, for phonons belonging to the S^3 branch in the two membranes, not shown in the main article. A decrease as large as an order of magnitude is found in the NS sample. While in both samples τ decreases with ν, the slope of this decrease is reduced in the NS sample. However, since we have only two points we cannot further comment on the dependence on ν. In table II, the mean free path ℓ is also calculated for the phonons belonging to branches S^2 and S^3, using the velocities obtained from the analytically calculated Lamb dispersions. Within error bars, the velocity at these frequencies does not change with nanostructuration, thus the mean free path reduction reflects the one of the lifetime. The smallest mean free path is found at λ_{ph}=55.8 nm, for which it is ℓ =0.08(1) μm.

Sample	Branch	$\boldsymbol{\lambda_{ph}}$(nm)	A	$\boldsymbol{\nu}$(GHz)	$\boldsymbol{\tau}$(ps)	$\mathbf{A_{th}}$	$\boldsymbol{\tau_{th}}$(ps)	v(Km/s))	$\boldsymbol{\ell}$($\boldsymbol{\mu}$m)
U	S^2	55.8	3.66(8)	168.3(1)	138(8)	7.99(6)	156(3)	7.2(1)	1.00(6)
U	S^2	83.7	3.13(5)	118.7(1)	270(10)	4.20(4)	380(10)	8.5(1)	2.3(1)
U	S^2	109.7	1.89(5)	96.10(2)	330(20)	3.09(3)	570(20)	6.5(1)	2.1(1)
U	S^3	55.8	2.51(7)	193.3(1)	170(8)			6.5(1)	1.13(5)
U	S^3	83.7	0.32(3)	159.6(1)	1000(300)			5.7(1)	5(3)
NS	S^2	55.8	1.6(4)	169.7(7)	42(9)	8.65(5)	140(2)	7.2(1)	0.30(7)
NS	S^2	83.7	1.45(5)	117.8(1)	100(6)	2.64(3)	330(10)	8.5(1)	0.85(5)
NS	S^2	109.7	1.47(5)	96.3(1)	90(5)	3.02(2)	420(10)	6.5(1)	0.59(3)
NS	S^3	55.8	2.2(3)	197(1)	12(2)			6.5(1)	0.08(1)
NS	S^3	83.7	0.18(4)	158.8(3)	160(70)			5.7(1)	0.9(4)
NS	new	55.8	1.6(2)	103.4(3)	37(4)				
NS	new	55.8	0.7(3)	175.1(2)	300(200)				
NS	new	55.8	0.4(4)	122.4(4))	110(50)				

Supplementary Table II. **Fit parameters for the uniform and nanostructured membranes. U and NS stand for Uniform and Nanostructured.** A_{ph},ν_{ph},τ_{ph} **are amplitude, frequency and lifetime for the give phonon,** A_{th} **and** τ_{th} **are the thermal relaxation amplitude and characteristic time, reported only once for a given sample and value of** L_{TG}**.** Velocities are calculated as the tangent to the analytical Lamb dispersions, and the mean free path as $\ell = v\tau_{ph}$.

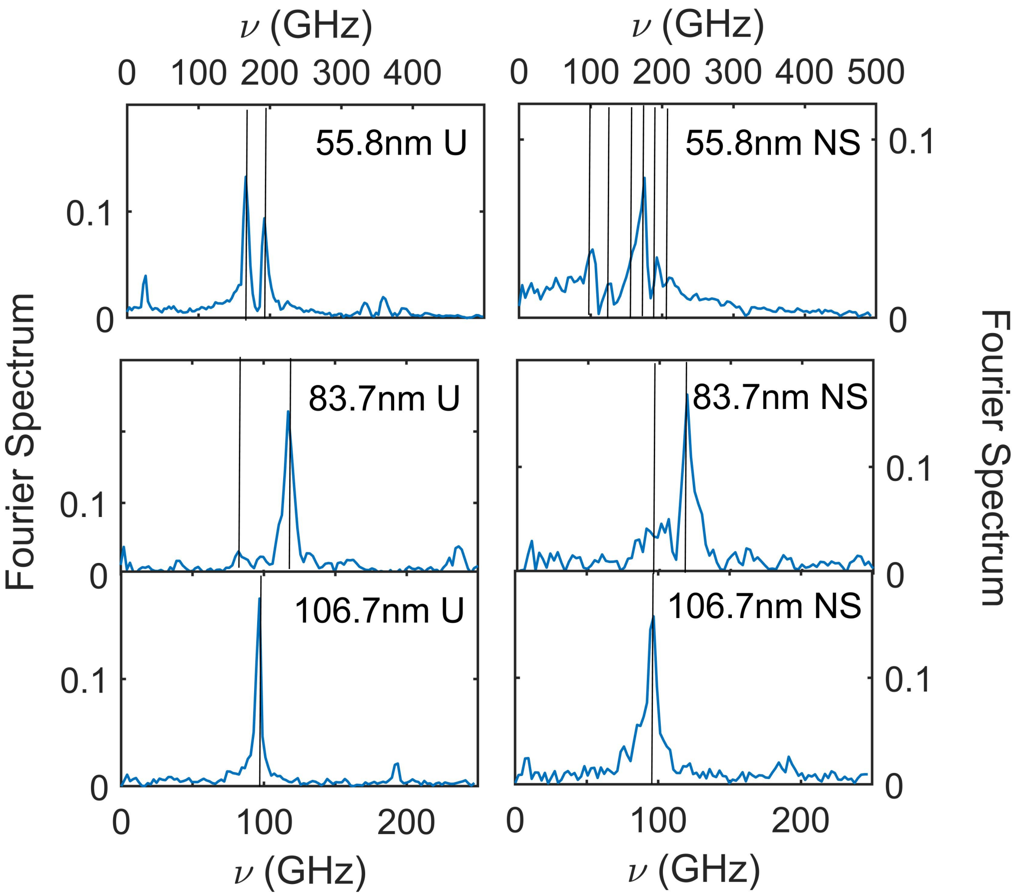

Supplementary Figure 1. **Fourier transforms of the EUV TG signals reported in the main article.** The Fourier Transforms of the experimental waveforms are reported for the uniform (U, left column) and nanostructured (NS, right column) samples at the investigated TG periods. Vertical lines indicate the main peaks identified for each case.

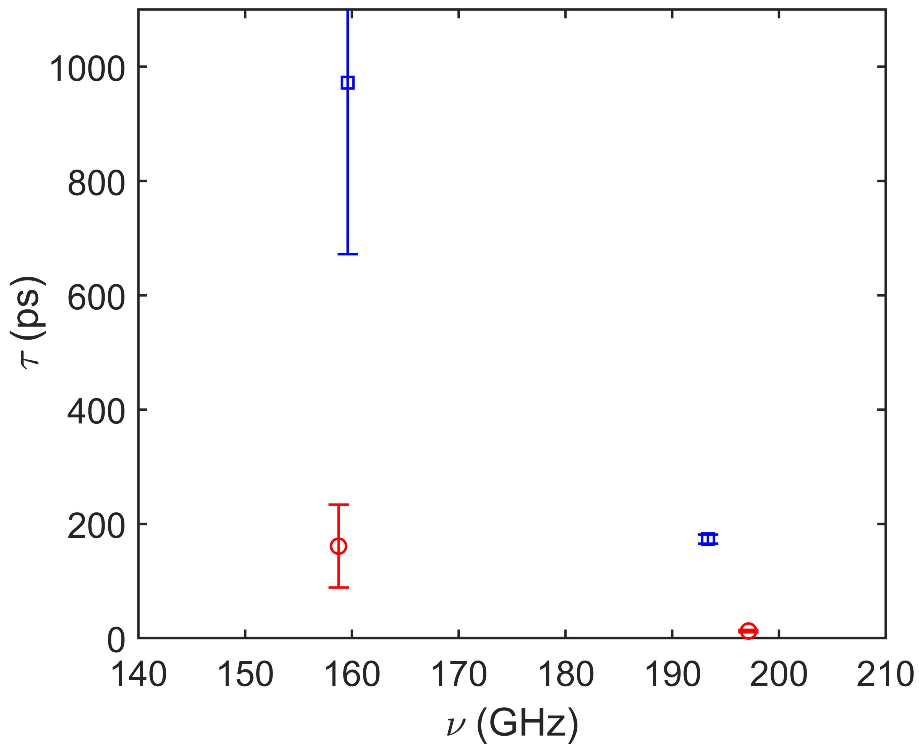

Supplementary Figure 2. **Experimental phonon lifetime for the branch** S^3 The values of τ, as obtained from the fit of the EUV TG waveforms, are reported for the uniform (blue squares) and the nanostructured (red circles) membranes as a function of ν; error bars come from the fit uncertainty.

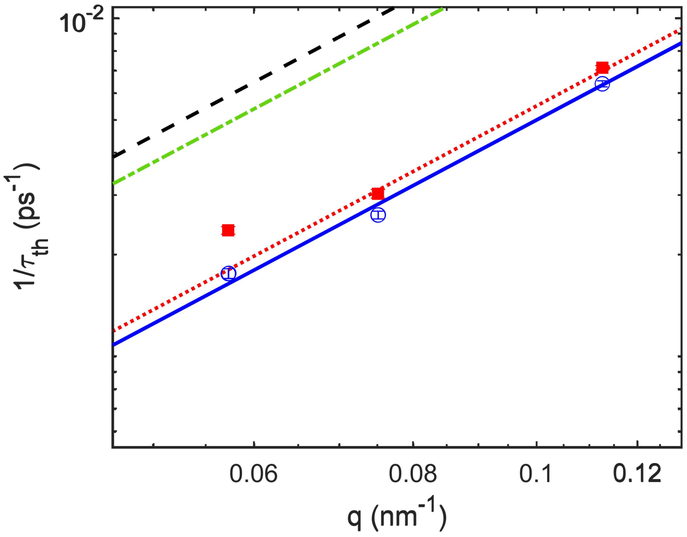

Supplementary Figure 3. **The thermal relaxation rate as a function of** q. The thermal relaxation rate, as obtained from the fit of our experimental spectra, is reported as a function of k in logarithmic scale for the uniform (blue circles) and nanostructured (red squares) membranes. Error bars come from the uncertainty of the fit of the experimental spectra. Blue solid and red dotted lines are the fit giving, respectively, $D^U_{th} = (0.5 \pm 0.05)\mathrm{m}^2\mathrm{s}^{-1}$ and $D^{NP}_{th} = (0.55 \pm 0.05)\mathrm{m}^2\mathrm{s}^{-1}$. Dashed black and dot-dashed green lines are the expected trends using the thermal diffusivity measured in 1.4μm and 600 nm thick membranes, respectively [4].

III. THERMAL RELAXATION

The thermal relaxation rate, as extracted from the best fit of EUV TG waveforms with Eq. (1) in the main text, is reported in Supplementary Fig. 3 as a function of k for both the uniform and nanostructured membranes

Given the pump absorption lengths reported in table I of the Methods section in the main text, thermal transport takes place both cross-plane, from the front surface illuminated by the laser towards the back one, and in-plane, from hot to cold interference fringes of the TG. In our experimental geometry we are mostly sensitive to the in-plane direction, where the equivalent heat transport distance is $L_{th} = L_{TG}/\pi$ [3].

The heat diffusion theory, which is valid at macroscopic length scale (*i.e.* for values of L_{th} significantly larger than the average phonon mean free path), predicts a k^2 dependence of the thermal relaxation rate:

$$\tau_{th}^{-1} = D_{th} k^2 \quad (1)$$

with D_{th} the thermal diffusivity, which can be calculated from the thermal conductivity k_T, C and ρ: $D_{th} = k_T/\rho C$. For bulk SiN, using the literature values $k_T = 3\mathrm{Wm}^{-1}\mathrm{K}^{-1}$, $C = 700$ J $\mathrm{Kg}^{-1}\mathrm{K}^{-1}$ [5] and our nominal density, one would get $D_{th} = 1.48\mathrm{x}10^{-6}$ $\mathrm{m}^2\mathrm{s}^{-1}$, in fair agreement with values reported in thick membranes [4]. Despite in our case L_{th} is expected to be comparable to the phonon mean free paths [6, 7], we clearly observe a diffusive thermal transport ($\tau_{th}^{-1} \propto k^2$), differently from what reported in other (crystalline) materials [8, 9]. However, our estimated value of $D_{th} \sim (0.5 \pm 0.05)10^{-6}$ $\mathrm{m}^2\mathrm{s}^{-1}$ is significantly smaller than the bulk one, confirming previous reports of D_{th} in amorphous SiN membranes of 50 and 100 nm thickness at comparable values of L_{th} [10, 11]. It is worth noticing in these latter works, the fluence, and thus the sample temperature, was much higher: this points to a weak temperature dependence of D_{th} in this material. Surprisingly, nanostructuration does not change significantly τ_{th}^{-1} in the probed L_{th} range (*i.e.* 18-35 nm): we still observe a diffusive behavior, with only a 10% increase in D_{th}. The lack of substantial change is in agreement with previous reports on macroscopic thermal conductivity on similar membranes, for which a sizable reduction was observed only for small necks values [7]. It's worth noticing that, in Supplementary Fig. 3, the smallest k point is clearly out of the trend, indicating a larger value of D_{th} at $L_{TG} = 109.6$ nm. As this data point corresponds to the shortest absorption length for the pump laser, the effects of the finite penetration depth of the EUV pulses in cross-plane heat transport is expectedly more sizable. Even if we are mostly probing in-plane thermal transport, we cannot exclude a cross-talking between heat fluxes along these two directions. However, with the data in hand, we cannot draw any conclusion on this aspect; further studies are needed to assess the nanoscale thermal relaxation dynamics in these membranes.

IV. THEORETICAL RESULTS AND ANALYSIS

To analyse the wavepacket propagation in our sample, we have measured the envelope of the kinetic energy induced in the system by the propagation of the wavepacket in the x direction (see Supplementary Fig. 5 in the main article), parallel to the EUV TG wavevector, averaged over both y and z directions. The energy envelope is defined for each excitation frequency ν as

$$P_\nu(x) = \max_t E_k(x,t) \tag{2}$$

where $E_k(x,t)$ is the instantaneous kinetic energy supported by the frame located in x with width $\Delta x = 2$ Å.

Since the simulations are performed at constant energy (no damping term in the numerical scheme), no attenuation would be observed in a uniform membrane, and the one arising in the nanostructured material is due to a redistribution of the kinetic energy in directions different from the one (x-axis) of the initially excited wavepacket, which we call propagation direction, so that an effective reduction of the kinetic energy $E_{kin}(x,t)$ along x appears. It has been shown [12, 13] that, depending on the nanostructure geometrical and elastic parameters, and on the wavepacket wavelength, different scenarios can arise. If the wavepacket keeps its propagative nature, a global exponential attenuation of $P_\nu(x)$ along the propagation direction x, similar to a Beer-Lambert law, is observed:

$$P_\nu(x) \propto \exp(-x/\ell_{env}(\omega)) \tag{3}$$

with ℓ_{env} the envelope mean free path. However, if scattering is predominantly diffusive, the observed behavior will be [14]

$$P_\nu(x) \propto 1/x \tag{4}$$

Finally, for materials very different on the two sides of an interface, the attenuation can be extremely efficient leading to an energy localization.

Supplementary Fig. 4 reports the envelopes for all the simulated wavepackets, divided in two panels: from 58 to 102 nm wavelengths on the left hand side and from 102 to 502 nm on the right hand side. The representation is in a semi-logarithmic scale, so that a propagative behavior can be recognized by a linear dependence of E_{kin} on the distance (x) (see Eq. 3), with a slope which is directly the inverse of ℓ_{env} for the envelope of the considered wavepacket [12]. It is evident how data are smoother at short λ_{ph}'s and becomes noisy at larger λ_{ph}'s. In addition, for $\lambda_{ph} < 102$ nm, periodic peaks can be identified, which progressively disappear on increasing λ_{ph} for reappearing again above $\lambda_{ph} = 402$ nm. They may have two origins: i) the mass difference between regions with and without holes (which leads to a periodic mass profile function along x) and ii) constructive interference between the main peak and the backreflected one. To account for this trend, we fit the curves in Supplementary Fig. 4 with an exponential decay plus a sinusoidal oscillation wherever peaks are well distinguishable, and only the exponential decay elsewhere:

$$E_{kin}(x)/E_{kin}(0) = e^{-x/\ell_{env}} + a\ sin(x/b + \phi) \tag{5}$$

with a, b and ϕ the amplitude, periodicity and phase of the oscillations. The slope progressively increases on increasing λ_{ph} up to 102 nm, and then becomes almost constant with λ_{ph}, starting to decrease again at $\lambda_{ph} = 402$ nm. As such, $\lambda_{ph} \approx$100 nm marks the transition between different regimes: above $\approx$ 100 nm and below 402 nm the slope does not change significantly, but also the signal appears noisy and the periodic peaks are definitely disrupted. Such disrpution is likely related to the longer spatial extension of the wavepacket. The temporal coherence length of the wavepacket is $t_0 = \frac{3}{2\nu} = \frac{3}{2}T$ with T the period (see Methods section in the main article), which gives wave-packets with 9 periods, with a total time extension of about $6t_0$. If we only get the half maximum width of the wave-packet, such length is about 3.5 periods, thus $\Delta t_{FWHM} = 3.5\frac{2}{3}t_0$. Using this temporal extension and the velocity (whose derivation is described later in this section), we have calculated the spatial extension and reported it in table III. It may be seen that this latter, which represents an estimation of the coherence length, becomes definitely larger than the neck for $\lambda_{ph} > 102$ nm; as such, the overlap between main and backreflected peak spreads all over the neck and no well-defined maximum can be identified. The situation for $\lambda_{ph} > 402$ nm is different: while the coherence length is much larger than the neck, we can still clearly identify peaks. These peaks are most likely a combined effect of the mass difference between holey and uniform regions and the oscillations within the wavepacket, which has now a very large spatial extent.

In Supplementary Fig. 5 we report the mean free path ℓ_{env} obtained fitting the curves with Eq. 5, as well as the periodicity b for the wavelengths where oscillations are distinguishable. We first comment shortly on this latter

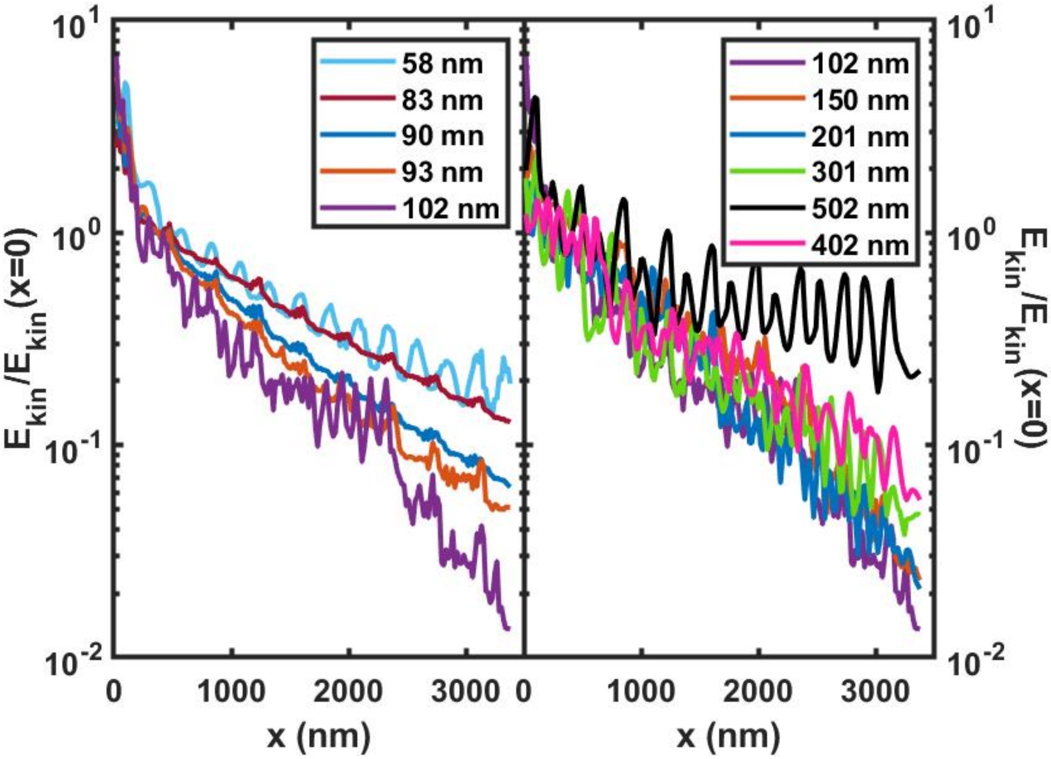

Supplementary Figure 4. **Simulated kinetic energy envelopes** The envelope of E_{kin} as a function of the x coordinate (*i.e.* parallel to the EUV TG wavevector) is reported for λ_{ph} ranging from 58 to 102 nm (figure on the left hand side) and form 102 to 502 nm (figure on the right hand side).

parameter: one would expect $b \sim 377$ nm, *i.e.* a periodicity reflecting the one of the pores lattice, as due to the mass difference between regions with and without holes . This is true for $83 \leq \lambda_{ph} \leq 100$ nm. Surprisingly, for $\lambda_{ph} = 58$ nm, we find $b = 251.1(6)$ nm, pointing to an energy periodically localized at the borders of the holes, where the peak and its backreflection can contructively interfere within the short coherence length of the wavepacket. $\lambda \geq 402$ nm have a periodicity half the one of the lattice. As the kinetic energy corresponds to the squared intensity of the generated wavepacket, the wavelength of the wavepacket oscillations here will be half the phonon wavelength, *i.e.* 201-251 nm. The oscillating signal is thus most likely the envelope of the wavepacket oscillations with the function representing the mass profile along x.

Coming now to the mean free path, a steep reduction settles in just above 84 nm, pointing to a sudden effect of the nanostructure for wavelengths between 84 and 102 nm, at which point the mean free path reaches a minimum, before starting increasing again above 201 nm. In order to understand what's happening, we look at the time dependence of the wavepacket, following the time evolution of the kinetic energy at 18 positions in the sample: 9 at x positions corresponding to the center of the holes and 9 at the center of the neck. We report it in Supplementary Fig. 6, where the wavepacket can be followed in time at the 18 positions through the different coloured curves, at all simulated wavelengths, in a time window longer than the time needed for the first pulse to travel through the whole sample. From a global look, it is evident that the only wavelength which propagates really well, with a single peak gradually loosing intensity because of attenuation, is $\lambda = 84$ nm. At all other wavelengths, at each position in the sample, we can observe secondary peaks, due to interference, appearing later in time and which, at longer travelled distances, may become even more intense than the first pulse. This can be better seen in Supplementary Fig. 7 and Supplementary Fig. 8 , where the temporal dependence is reported for all wavelengths at the same two central positions about 1300 nm from the beginning of the sample: at the center of the neck in the first case, and of the hole in the second. Here we can see that even at 84 nm there are actually interference peaks, which, however, remain well below the intensity of the primary pulse. This is not the case for the other wavelengths, except for $\lambda_{ph} = 502$ nm, where, despite the interference peaks are intense, they remain slightly weaker than the main one. It is worth noticing that the presence of clearly defined interference peaks is only possible for multiple scattering events which keep the phase of the wavepacket, otherwise we should have a non-structured background, as the one present at 58 nm after the

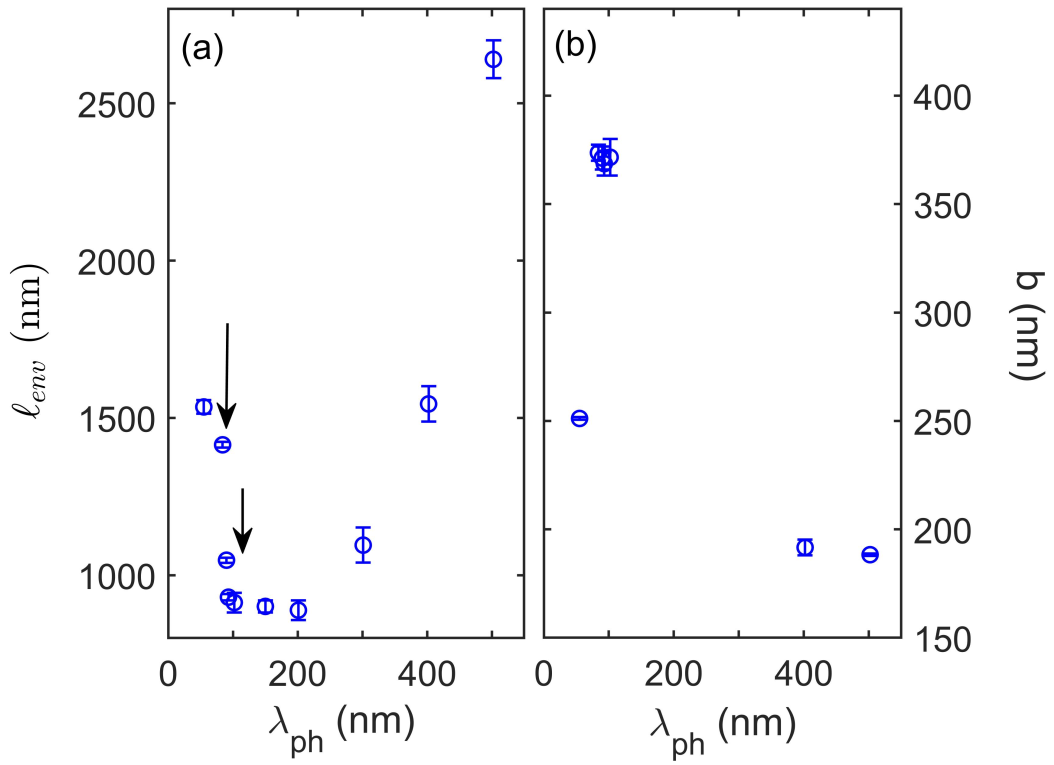

Supplementary Figure 5. **(a): Simulated wavepacket mean free path**. The value of ℓ_{env}, as obtained from fitting the E_{kin} envelopes with Eq. 5, are reported as a function of λ_{ph}. A steep decrease sets in above 84 nm (marked by an arrow) and leads to a shallow minimum around 150 nm. A second arrow marks the change of regime at ~ 100 nm from a decreasing mean free path with λ_{ph} to an almost constant mean free path (see text). **(b): Periodicity of local maxima in the kinetic envelope**: the parameter b from Eq. 5 is reported as a function of λ_{ph} for the signals where oscillations are distinguishable: $\lambda_{ph} \leq 102$ nm and $\lambda_{ph} \geq 402$ nm. For this latter range, this parameter results from the combined effect of the pulse oscillations and the mass modulation. Both in (a) and (b), error bars come from fit uncertainties.

second peak in Supplementary Figs. 7 and 8. As such, we are definitely in presence of a coherent interference.

Looking at these figures, we can better understand the envelopes of Supplementary Fig. 4, which show the maximum of the kinetic energy over all times (see Eq. 2): at long λ's, high intensity interference peaks appear, which may become more intense than the first peak, modifying the global slope of $P_\nu(x)$. As such, at small wavelengths, the envelope attenuation reflects the attenuation of the main wavepacket, but it won't be so at large wavelengths, as for a given position x, the maximum over all times can belong to a peak coming later in time and not the first one.

For the wavelength range experimentally investigated, the propagation remains quite smooth, so we have fit the amplitude of the first peak as a function of time to get the wavepacket lifetime (τ_{coh}) and compare it with our experimental data. From the position of the first peak as a function of time we can also get the wavepacket velocity, and then calculate the mean free path for the first peak, ℓ_{coh}. We have compared it with the one obtained from the envelope analysis, ℓ_{env} in Supplementary Fig. 6 of the main article, showing that at our experimental wavelengths the values are very close. Increasing wavelength above 102 nm and below 502 nm, the values of the mean free path obtained using the two approaches significantly differ: ℓ_{env} is systematically larger than ℓ_{coh}, due to the interference peaks which are, over all times, more intense than the first one and thus dominate the slope of $P_\nu(x)$. This means that there is an important constructive interference, which contributes to reducing the wavepacket lifetime, while increasing the energy which remains in the sample and propagates through the reflections. At λ_{ph}=502 nm, this is not true anymore: we start to be less sensitive to the effects of the nanostructure, as the wavelength becomes larger than both neck and pitch. In these conditions, the values of the mean free path obtained in both ways are again consistent.

Results are reported in Supplementary Table III for all wavelengths. In the main work, we have used the lifetime obtained from the temporal analysis of the first peak. If instead, we use the ones obtained from the envelope mean free path and the theoretical velocity, we find that the result does not significantly change: our experimental data are still well reproduced using directly these theoretical lifetimes together with the experimental ones from the uniform

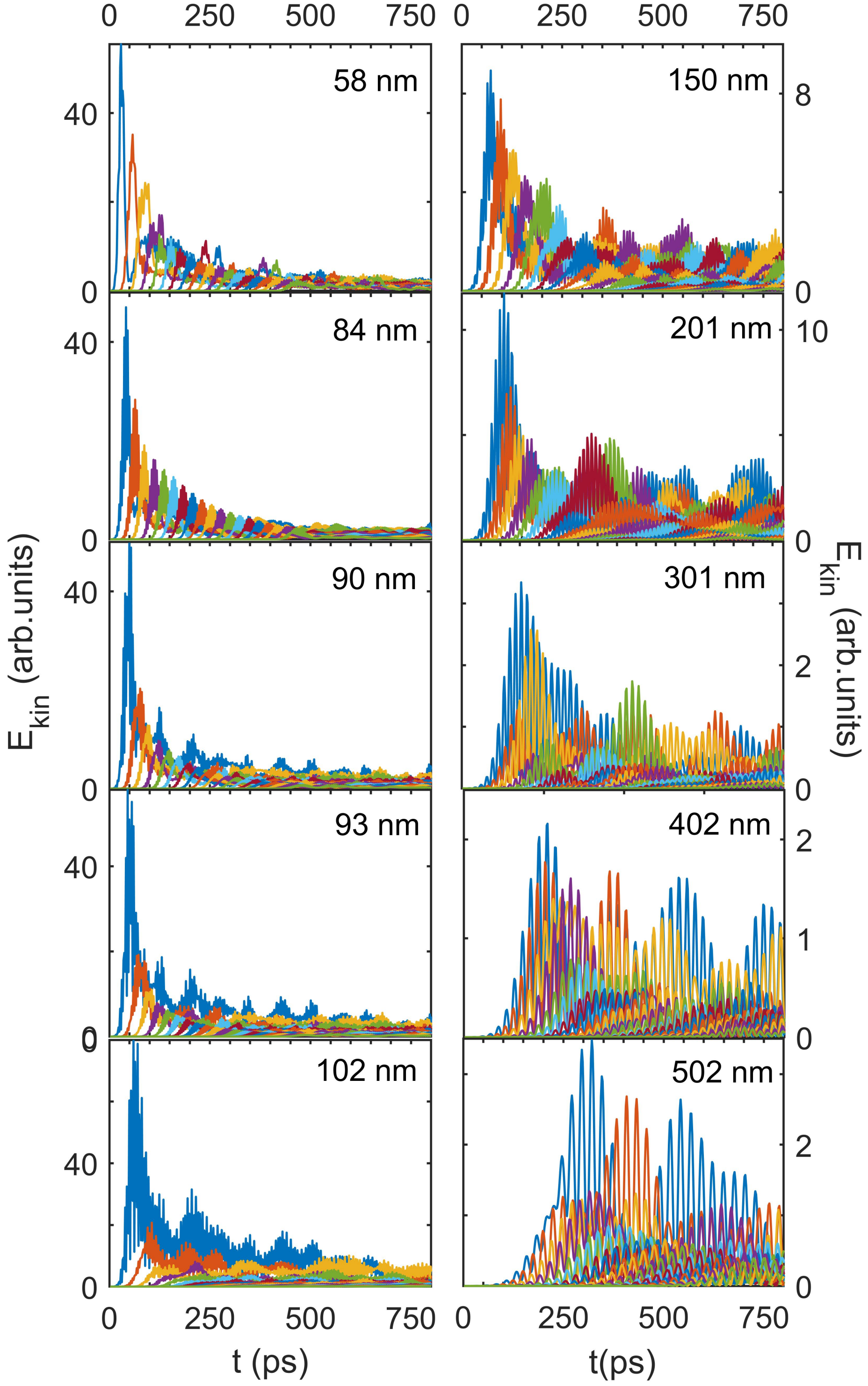

Supplementary Figure 6. **Time dependence of the kinetic energy over the 18 positions in the sample** The kinetic energy is followed as a function of time at 18 positions in the sample, corresponding to the centers of the necks and of the holes. Different colours identify the different positions. The first pulse can be identified and followed in its propagation through the whole sample at $\lambda = 58$ and 84 nm, above which wavelengths, the backrelected peaks start to become as important as the first peak in the center of the sample.

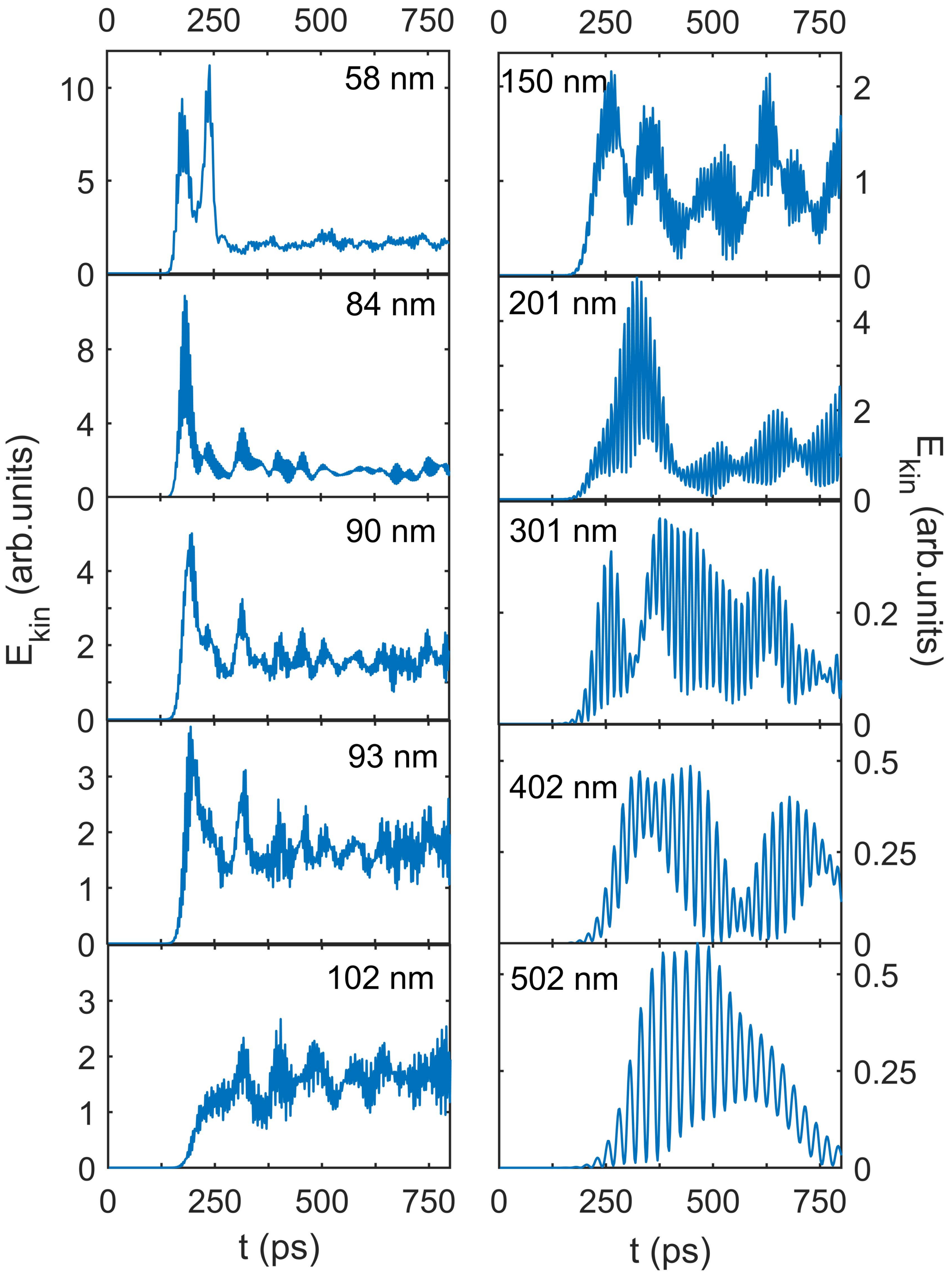

Supplementary Figure 7. **Time dependence of the kinetic energy** at the 7th position within the sample, corresponding to about 1300 nm from the beginning of the sample.

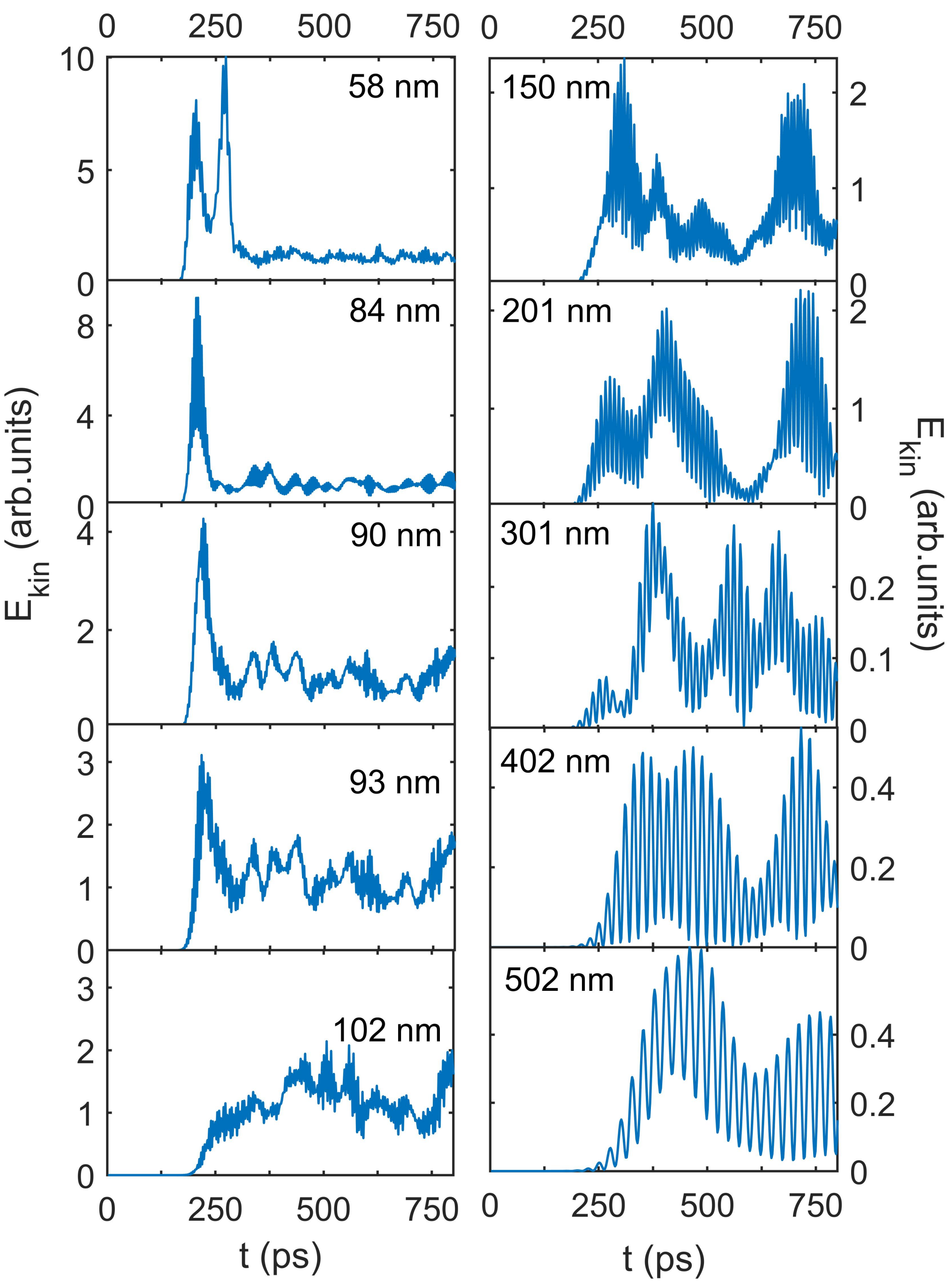

Supplementary Figure 8. **Time dependence of the kinetic energy** at the 8th position within the sample, corresponding to about 1300 nm from the beginning of the sample.

membrane.

One point of interest is the behavior observed for $\lambda_{ph} = 58$ nm: here we have a single peak later in time, which is more intense than the first one. It is likely due to the backreflected peak from the first interface, which constructively interferes with the incoming first peak. Still, the absence of other peaks would indicate that interferences with successive echos are destructive. Moreover, the arrival time of this peak is about 60 ps after the first one, which, at the velocity of this wavepacket, corresponds to a travelled distance of about 505 nm, *i.e.* twice the neck, which would be consistent with two backreflections between pores. The result is again a difference between ℓ_{env} and ℓ_{coh}. This could indicate the arising of another phenomenology for wavelengths shorter than the ones here probed, with the loss of interference effects except for specific wavelengths. More simulations are needed to understand this behavior and the role of coherent and incoherent contributions at shorter wavelengths.

Supplementary Table III. **Simulated wavepacket properties.** τ_{coh} is the lifetime of the main wavepacket obtained fitting its amplitude as a function of time. The velocity has been obtained fitting the position of the main wavepacket with respect to time. ℓ_{coh} is the mean free path calculated as $\ell_{coh} = v\tau_{coh}$. ℓ_{env} is the mean free path form the fit of the kinetic energy envelopes with Eq. 5, while L_c is the coherence length calculated from the temporal extension t_0 of the wavepacket and its velocity: $L_c = \Delta t_{FWHM} v$ with Δt_{FWHM} the time extension of the wavepacket at half its maximum intensity.

$\boldsymbol{\lambda_{ph}}$**(nm)**	$\boldsymbol{\nu}$**(GHz)**	$\boldsymbol{\tau_{coh}}$**(ps)**	**v(Km/s)**	$\boldsymbol{\ell_{coh}}$**(nm)**	$\boldsymbol{\ell_{env}}$**(nm)**	$L_c(nm)$
58	169.7	164(7)	7.737(1)	1270(50)	1530(20)	159.40(2)
83	117.8	174(2)	7.997(1)	1390(10)	1414(9)	237.60(3)
90	109.36	167(2)	7.867(1)	1320(20)	1048(8)	251.80(3)(1)
93	105.83	139(2)	7.8138(1)	1084(20)	930(10)	258.40(3)
102	96.3	139(8)	6.675(1)	930(60)	910(30	242.60(4)
150	65.6	100(10)	5.902(1)	570(80)	900(20)	314.90(5)
201	49.2	55(3)	7.850(1)	430(30)	890(30)	558.50(7)
301	32.8	36(6)	9.760(1)	350 (60)	1090(50)	1041.4(1)
402	24.6	99(9)	8.143(1)	810(70)	1540(60)	1158.5(1)
502	19.7	400(70)	6.518(1)	2600 (400)	2640(60)	1158.1(2)

V. EXPERIMENTAL PHONON LIFETIME ANALYSIS AND COHERENT CHARACTER

As reported in the main text, we have fitted the experimental phonon lifetime in the uniform membrane using two contributions, the anharmonic phonon-phonon scattering, modelled using the Akhiezer approach [15], and the boundary scattering from top and bottom surfaces, which depends on the roughness of these latter [16]. As for the nanostructured membrane we have added to these two contributions, the one from the nanostructure, as simulated, and which we have called τ_{coh}. This modelling could seem simplistic, since a more proper theoretical description of the experiment would imply considering roughness and anharmonicity in the calculations, as well as the finite penetration depth of the excitation. Still, its advantage is really the fact that, being the material ideal, with no roughness and no anharmonicity, the observed effect is clearly a coherent effect from the nanostructure geometry, leaving no ambiguity on its origin.

In Supplementary Table IV we report all the fitted contributions for the two samples. It may be seen that in the nanostructured one the coherent lifetime is longer than the total incoherent contribution at $\lambda_{ph} = 55.8$ nm, by almost a factor of 2, but then it decreases, becoming smaller than the incoherent contribution already at $\lambda_{ph} = 83.7$ nm (about 75% of it) and it is half the incoherent contribution at $\lambda_{ph} = 109.7$ nm, clearly dominating the total phonon lifetime.

In order to quantify the coherent character, we calculate the relative coherent contribution to the total phonon decay rate:

$$\chi = \frac{\Gamma_{coh}}{\Gamma_{coh} + \Gamma_{ph-ph} + \Gamma_b} \tag{6}$$

where $\Gamma_{coh} = \tau_{coh}^{-1}$, $\Gamma_{ph-ph} = \tau_{ph-ph}^{-1}$ and $\Gamma_b = \tau_b^{-1}$. We found that coherent mechanisms account for 36(2), 57.6(5) and 65(1)% of the total phonon attenuation for λ_{ph} =55.8, 83.7 and 109.7 nm, respectively, confirming their increasing relevance at our largest value of λ_{ph}.

Uniform membrane

$\boldsymbol{\lambda_{ph}}$**(nm)**	$\boldsymbol{\tau_{ph-ph}}$**(ps)**	$\boldsymbol{\tau_b}$**(ps)**	$\boldsymbol{\tau_{inc}}$**(ps)**
55.8	190(40)	230(30)	100(20)
83.7	700(100)	440(70)	270(40)
109.8	340(60)	1000(100)	250(40)

Nanostructured membrane

$\boldsymbol{\lambda_{ph}}$**(nm)**	$\boldsymbol{\tau_{ph-ph}}$**(ps)**	$\boldsymbol{\tau_b}$**(ps)**	$\boldsymbol{\tau_{inc}}$**(ps)**	$\boldsymbol{\tau_{coh}}$**(ps)**	$\boldsymbol{\chi}$(%)
55.8	160(3)	218(5)	92(2)	164(7)	36(2)
83.7	545(9)	418(9)	236(5)	174(2)	57.6(5)
109.7	347(6)	933(20)	253(5)	139(2)	65(1)

Supplementary Table IV. **Contributions to phonon lifetime as obtained from the fit of the experimental data.** τ_{ph-ph} is the phonon lifetime due to phonon-phonon scattering, τ_b is the phonon lifetime due to boundary scattering. τ_{inc} is the total phonon lifetime for these two contributons. $\tau_{coh}$is the coherent contribution to phonon lifetime as simulated by finite elements calculations.

Supplementary References

[1] L. Foglia, R. Mincigrucci, A. Maznev, G. Baldi, F. Capotondi, F. Caporaletti, R. Comin, D. D. Angelis, R. Duncan, D. Fainozzi, G. Kurdi, J. Li, A. Martinelli, C. Masciovecchio, G. Monaco, A. Milloch, K. Nelson, C. Occhialini, M. Pancaldi, E. Pedersoli, J. Pelli-Cresi, A. Simoncig, F. Travasso, B. Wehinger, M. Zanatta, and F. Bencivenga, Extreme ultraviolet transient gratings: A tool for nanoscale photoacoustics, Photoacoustics **29**, 100453 (2023).

[2] R. Mincigrucci, L. Foglia, D. Naumenko, E. Pedersoli, A. Simoncig, R. Cucini, A. Gessini, M. Kiskinova, G. Kurdi, N. Mahne, M. Manfredda, I. Nikolov, E. Principi, L. Raimondi, M. Zangrando, C. Masciovecchio, F. Capotondi, and F. Bencivenga, Advances in instrumentation for FEL-based four-wave-mixing experiments, Nuclear Instruments and Methods in Physics Research Section A: Accelerators, Spectrometers, Detectors and Associated Equipment **907**, 132 (2018).

[3] A. Vega-Flick, R. A. Duncan, J. K. Eliason, J. Cuffe, J. A. Johnson, J.-P. M. Peraud, L. Zeng, Z. Lu, A. A. Maznev, E. N. Wang, J. J. Alvarado-Gil, M. Sledzinska, C. M. S. Torres, G. Chen, and K. A. Nelson, Thermal transport in suspended silicon membranes measured by laser-induced transient gratings, AIP Advances **6**, 121903 (2016).

[4] X. Zhang and C. P. Grigoropoulos, Thermal conductivity and diffusivity of free-standing silicon nitride thin films, Review of scientific instruments **66**, 1115 (1995).

[5] H. Ftouni, C. Blanc, D. Tainoff, A. D. Fefferman, M. Defoort, K. J. Lulla, J. Richard, E. Collin, and O. Bourgeois, Thermal conductivity of silicon nitride membranes is not sensitive to stress, Physical Review B **92**, 10.1103/physrevb.92.125439 (2015).

[6] R. Sultan, A. D. Avery, J. M. Underwood, S. J. Mason, D. Bassett, and B. L. Zink, Heat transport by long mean free path vibrations in amorphous silicon nitride near room temperature, Physical Review B **87**, 10.1103/physrevb.87.214305 (2013).

[7] N. Tambo, Y. Liao, C. Zhou, E. M. Ashley, K. Takahashi, P. F. Nealey, Y. Naito, and J. Shiomi, Ultimate suppression of thermal transport in amorphous silicon nitride by phononic nanostructure, Science Advances **6**, 10.1126/sciadv.abc0075 (2020).

[8] D. Naumenko, R. Mincigrucci, M. Altissimo, L. Foglia, A. Gessini, G. Kurdi, I. Nikolov, E. Pedersoli, E. Principi, A. Simoncig, M. Kiskinova, C. Masciovecchio, F. Capotondi, and F. Bencivenga, Thermoelasticity of nanoscale silicon carbide membranes excited by extreme ultraviolet transient gratings: Implications for mechanical and thermal management, ACS Applied Nano Materials **2**, 5132 (2019).

[9] J. A. Johnson, A. A. Maznev, J. Cuffe, J. K. Eliason, A. J. Minnich, T. Kehoe, C. M. S. Torres, G. Chen, and K. A. Nelson, Direct measurement of room-temperature nondiffusive thermal transport over micron distances in a silicon membrane, Physical Review Letters **110**, 10.1103/physrevlett.110.025901 (2013).

[10] F. Bencivenga, R. Mincigrucci, F. Capotondi, L. Foglia, D. Naumenko, A. A. Maznev, E. Pedersoli, A. Simoncig, F. Caporaletti, V. Chiloyan, R. Cucini, F. Dallari, R. A. Duncan, T. D. Frazer, G. Gaio, A. Gessini, L. Giannessi, S. Huberman, H. Kapteyn, J. Knobloch, G. Kurdi, N. Mahne, M. Manfredda, A. Martinelli, M. Murnane, E. Principi, L. Raimondi, S. Spampinati, C. Spezzani, M. Trovò, M. Zangrando, G. Chen, G. Monaco, K. A. Nelson, and C. Masciovecchio, Nanoscale transient gratings excited and probed by extreme ultraviolet femtosecond pulses, Science Advances **5**, 10.1126/sciadv.aaw5805 (2019).

[11] A. Milloch, R. Mincigrucci, F. Capotondi, D. D. Angelis, L. Foglia, G. Kurdi, D. Naumenko, E. Pedersoli, J. S. Pelli-Cresi, A. Simoncig, B. Wehinger, C. Masciovecchio, and F. Bencivenga, Nanoscale thermoelasticity in silicon nitride membranes: Implications for thermal management, ACS Applied Nano Materials **4**, 10519 (2021).

[12] H. Luo, A. Gravouil, V. Giordano, and A. Tanguy, Thermal transport in a 2d nanophononic solid: Role of bi-phasic materials properties on acoustic attenuation and thermal diffusivity, Nanomaterials **9**, 1471 (2019).

[13] H. Luo, Y. Ren, A. Gravouil, V. M. Giordano, Q. Zhou, H. Wang, and A. Tanguy, Role of a fractal shape of the inclusions on acoustic attenuation in a nanocomposite, APL Materials **9**, 081109 (2021).

[14] Y. M. Beltukov, D. A. Parshin, V. M. Giordano, and A. Tanguy, Propagative and diffusive regimes of acoustic damping in bulk amorphous material, Physical Review E **98**, 10.1103/physreve.98.023005 (2018).

[15] Y. Liao and J. Shiomi, Akhiezer mechanism dominates relaxation of propagons in amorphous material at room temperature, Journal of Applied Physics **130**, 10.1063/5.0050159 (2021).

[16] J. M. Ziman, *Electrons and Phonons: The Theory of Transport Phenomena in Solids* (Oxford University Press, 1960).
